# The Association Between the Triglyceride-to-High-Density Lipoprotein Cholesterol Ratio and the Risk of Progression to Diabetes From Prediabetes: A 5-year Cohort Study in Chinese Adults

**DOI:** 10.3389/fendo.2022.947157

**Published:** 2022-07-18

**Authors:** Yanfei Sun, Zhibin Wang, Zhiqiang Huang, Haofei Hu, Yong Han

**Affiliations:** ^1^ Department of Trauma Center/Burns, The First Affiliated Hospital of China Medical University, Shenyang, China; ^2^ Department of Emergency, Shenzhen Second People’s Hospital, Shenzhen, China; ^3^ Department of Nephrology, Shenzhen Second People’s Hospital, Shenzhen, China

**Keywords:** dyslipidemia, diabetes, nonlinear, prediabetes, smooth curve fitting

## Abstract

**Objective:**

Evidence regarding the relationship between the triglyceride-to-high-density lipoprotein cholesterol (TG/HDL-c) ratio and the risk of progression from prediabetes to diabetes remains limited. The purpose of our study was to investigate the relationship between the TG/HDL-C ratio and incident diabetes in prediabetic patients.

**Methods:**

This retrospective cohort study covered 32 regions and 11 cities in China and consecutively and non-selectively collected data from 15,017 patients with prediabetes who had received a health check from 2010 to 2016. Data were obtained from the DATADRYAD database (www.datadryad.org). The Cox proportional-hazards regression model with cubic spline functions and smooth curve fitting (cubic spline smoothing) was used to explore the non-linear relationship between the baseline TG/HDL-c ratio and the risk of diabetes in patients with prediabetes. In addition, we performed a series of sensitivity and subgroup analyses.

**Results:**

The mean age of the included individuals was 50.95 ± 13.48 years, and 9,745 (64.51%) were men. The median (interquartile range) TG/HDL-c ratio was 1.09 (0.69–1.72). During a median follow-up time of 3.05 years, 1,731 (11.46%) patients had a final diagnosis of diabetes. The analysis after adjusting for covariates showed that the TG/HDL-c ratio was positively related to incident diabetes in patients with prediabetes (HR = 1.111, 95% CI 1.061–1.164). Participants with the highest TG/HDL-c ratio (Q4) had higher diabetes incidence rates than those with the lowest TG/HDL-c ratio (Q1) (P < 0.001 for the trend). There was a non-linear relationship between the TG/HDL-c ratio and the risk of diabetes, and the inflection point of the TG/HDL-c ratio was 1.415. The effect sizes (HR) on the left and right sides of the inflection point were 1.336 (95% CI: 1.134–1.573) and 1.055 (95% CI: 0.988–1.126), respectively. The sensitivity analysis demonstrated the robustness of these results.

**Conclusion:**

This study demonstrates a positive, non-linear relationship between the TG/HDL-c ratio and the risk of diabetes in Chinese patients with prediabetes. Aggressive intervention from a treatment perspective is required to lower the TG/HDL-c ratio below the inflection point (1.415) by lowering TG or increasing HDL-c levels.

## Introduction

Diabetes mellitus (DM) is a complex metabolic disorder that affects hundreds of millions of people worldwide and causes significant global ill health and economic burdens (1, 2). According to the International Diabetes Federation (IDF), the global diabetes prevalence in 20–79-year-olds in 2021 was estimated to be 10.5% (536.6 million people), rising to 12.2% (783.2 million) in 2045 (1). Diabetes is related to relatively specific microvascular complications affecting the eyes, nerves, and kidneys as well as an increased risk of cardiovascular disease (3–5). Global diabetes-related health expenditures were estimated at 966 billion USD in 2021 and are projected to reach 1,054 billion USD by 2045 (1).

Prediabetes refers to an intermediate stage of dysglycemia along a continuum from normoglycemia to diabetes. It generally reflects the presence of impaired fasting glucose or glucose tolerance. The IDF estimated that approximately 374 million adults had prediabetes in 2017, with a global prevalence of 7.7% (2). IDF projections indicate that the number of adults with prediabetes will reach 548 million by 2045, corresponding to 8.4% of the world’s adult population (6). According to a recent national cross-sectional survey, the prevalence of prediabetes among Chinese adults has reached 35.7% (7). The annual risk of developing diabetes in people with prediabetes is 5%–10%, and up to 70% eventually develop diabetes (8). Prediabetes is often considered to be a warning sign. Nevertheless, most individuals with prediabetes are unaware of this metabolic abnormality and ignore its importance. Therefore, it is particularly important to know the risk factors for diabetes in patients with prediabetes and provide timely intervention to prevent or delay the incidence of diabetes and its complications.

Dyslipidemia refers to abnormal blood lipid levels characterized by high levels of low-density lipoprotein cholesterol (LDL-c) and low levels of high-density lipoprotein cholesterol (HDL-c) and triglycerides (TG) (9). Any type of dyslipidemia, alone or in combination, is associated with an increased probability of diabetes (10). Recently, an atherogenic dyslipidemia parameter, namely, the triglyceride-to-high-density lipoprotein cholesterol (TG/HDL-c) ratio, has been thought to be related to insulin resistance (IR), cardiovascular events, incident hypertension, and fatty liver (11–13). Some studies have explored the association between the TG/HDL-c ratio and the incidence of diabetes. Their findings suggest a positive association between the TG/HDL-c ratio and diabetes, but the effect sizes of these results are inconsistent (14–17). In addition, previous studies investigating the association of the TG/HDL-c ratio with diabetes included the general population. Their relationship has not been reported in patients in the prediabetic stage, a population at high risk of developing diabetes. Therefore, we performed a retrospective cohort study using published data to determine the relationship between the TG/HDL-c ratio and the risk of progression from prediabetes to diabetes.

## Methods

### Study Design

This study used a retrospective cohort study design, and the data were obtained from a retrospective cohort study previously undertaken by Chinese researchers (Chen et al.) from a computerized database in China. The target-independent variable was the TG/HDL-c ratio at baseline. The outcome variable was diabetes (DM) (dichotomous variable: 0 = non-DM, 1 = DM) (18).

### Data Source

Raw data were obtained from the DATADRYAD database (www.datadryad.org) provided by Chen, Ying et al. (18); data were from a population-based cohort study which investigated the association of body mass index and age with incident diabetes in Chinese adults (called the Dryad Dataset, https://doi.org/10.5061/dryad.ft8750v). Under Dryad’s terms of reference, researchers could use these data for secondary analyses without violating the authors’ rights (18).

### Study Population

The original researchers extracted data from a computerized database established by the Rich Healthcare Group in China, which includes all medical records of participants who received a health check from 2010 to 2016, covering 32 regions and 11 cities in China (18). The original study was approved by the Rich Healthcare Group review board, and information was retrieved retrospectively. The institutional ethics committee did not require any study approval or informed consent for this retrospective study (18). Therefore, this secondary analysis did not require ethical approval. The original study was conducted in accordance with the Declaration of Helsinki. All methods were performed following the relevant guidelines and regulations, including a statement in the Declaration section. So did this secondary analysis.

**Figure 1:**
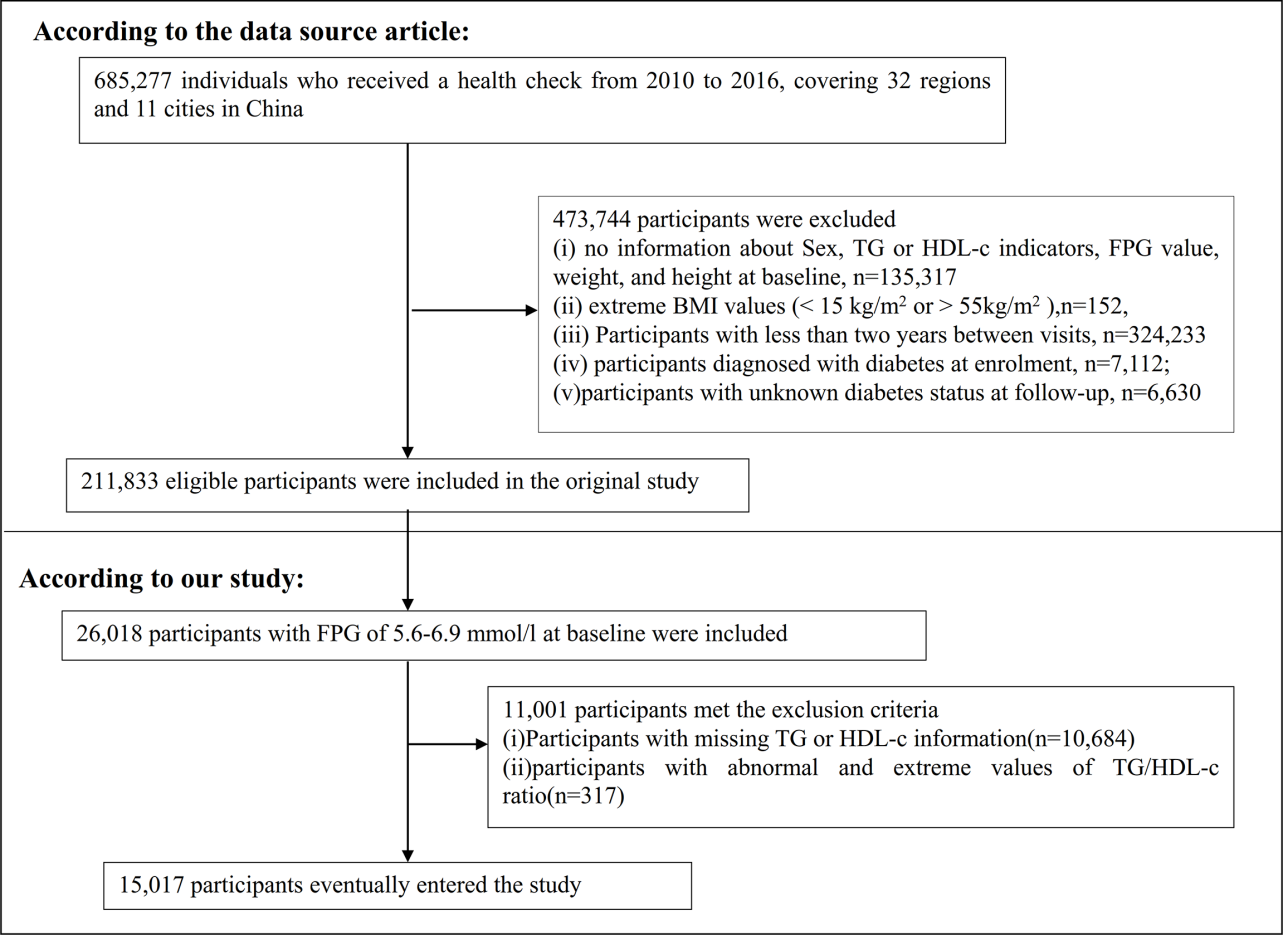
Flowchart of study participants.

The original study initially included 685,277 individuals who were at least 20 years old and had undergone at least two health checkups. In total, 473,744 participants were excluded from the original study. Ultimately, 211,833 individuals were included in the analysis of the original study. Exclusion criteria for the original study were as follows: (i) no information about sex, TG, or HDL-c indicators, fasting plasma glucose (FPG) value, weight, and height at baseline; (ii) extreme BMI values (<15 kg/m^2^ or >55 kg/m^2^); (iii) less than 2 years between visits; (iv) diagnosed with diabetes at enrolment; and (v) unknown diabetes status at follow-up (18). In the current study, we first included 26,018 participants with FPG of 5.6–6.9 mmol/l at baseline. According to the American Diabetes Association 2021 criteria, prediabetes is defined as an FPG level of between 5.6 and 6.9 mmol/l (19). We excluded participants with missing TG or HDL-c information (n = 10,684) and those with abnormal and extreme TG/HDL-c ratios (three standard deviations greater or less than three standard deviations from the mean) (n = 317) (20). Ultimately, 15,017 participants were included in the secondary analysis. **Figure 1** describes the participant-selection process.

### Data Collection

In the original study, trained investigators obtained baseline information through standard questionnaires, including the demographic characteristics (age and sex), lifestyle (smoking and drinking status), and family history of diabetes. BMI was calculated as weight in kilograms divided by height in meters squared. Standard mercury sphygmomanometers were used to measure blood pressure. Fasting venous blood samples were collected after at least 10 h of fasting at each visit. Plasma glucose, TG, TC (total cholesterol), HDL-c, and LDL-c levels were measured using an autoanalyzer (Beckman 5800) (18).

### Variables

The TG/HDL-c ratio was recorded as a continuous variable. The detailed process of defining the TG/HDL-c ratio was described: TG/HDL-c ratio = triglyceride divided by high-density lipoprotein cholesterol. It should be noted that TG and HDL-c units were both mmol/L.

### Outcome Measures

Our outcome variable of interest was incident diabetes (dichotomous variable: 0 = non-DM, 1 = DM). Incident diabetes was based on either self-report or fasting blood glucose ≥7.0 mmol/l at follow-up evaluation (18).

### Covariates

Covariates were selected according to our clinical experience and previous literature (10, 18, 21–26). The following variables were considered as covariates: (1) continuous variables: age, height, weight, body mass index (BMI), alanine aminotransferase (ALT), aspartate aminotransferase (AST), systolic blood pressure (SBP), diastolic blood pressure (DBP), blood urea nitrogen (BUN), serum creatinine (sCr), HDL-c, LDL-c, and TC; (2) categorical variables: sex, family history of diabetes, drinking status, and smoking status.

### Missing Data Processing

In observational research, missing data are regular occurrences that cannot be completely prevented (27). In our study, the number of participants with missing data for SBP, DBP, LDL-c, ALT, AST, BUN, sCr, drinking status, and smoking status was 5 (0.03%), 5 (0.03%), 26 (0.17%), 35 (0.23%), 8,120 (53.75%), 354 (2.34%), 113 (0.75%), 10,473 (69.33%), and 10,473 (69.33%), respectively. To mitigate the variation caused by missing variables, which cannot accurately reflect the statistical efficiency of the target sample throughout the modeling phase, this study used multiple imputations for missing data. The imputation model used linear regression and 10 iterations and included age, sex, BMI, SBP, DBP, ALT, AST, sCr, BUN, LDL-C, TC, smoking status, drinking status, and family history of diabetes. The missing data analysis procedures used missing-at-random (MAR) assumptions (27, 28).

### Statistical Analysis

We stratified the participants by TG/HDL-C ratio quartiles. The mean ± standard deviation (SD) (Gaussian distribution) or median (interquartile range) (skewed distribution) are reported for continuous variables, and frequencies and percentages are presented for categorical variables. We used χ^2^ (categorical variables) and one-way ANOVA (normal distribution), or Kruskal–Wallis H test (skewed distribution) to test for differences among different TG/HDL-c ratio groups.

To explore the link between the TG/HDL-c ratio and the risk of diabetes in patients with prediabetes, after collinearity screening, we used univariate and multivariable Cox proportional-hazards regression models, including a non-adjusted model (crude model: no covariates were adjusted), a minimally adjusted model (model I: adjusted age, sex, BMI), and a fully adjusted model (model II: adjusted age, sex, BMI, SBP, DBP, ALT, AST, sCr, smoking status, drinking status, and family history of diabetes). Effect sizes (HR) with 95% confidence intervals (CI) were calculated. We adjusted for confounding factors based on clinical experience, literature reports, and results of the univariate analysis and alluded to collinearity screening findings. Collinearity screening revealed that TC was collinear with the other variables and was not included in the final multivariable Cox proportional hazards regression equation.

We also employed a Cox proportional hazards regression model with cubic spline functions and smooth curve fitting to account for the non-linear connection between the TG/HDL-c ratio and diabetes risk in prediabetic participants. Moreover, a two-piecewise Cox proportional hazards regression model was employed to elucidate the non-linear relationship between the TG/HDL-c ratio and the risk of diabetes. Finally, a log-likelihood ratio test was used to determine the most appropriate model to describe the relationship between the TG/HDL-c ratio and the risk of diabetes in prediabetic patients.

Subgroup analyses across multiple subgroups were conducted using a stratified Cox proportional hazard regression model (sex, age, BMI, drinking status, smoking status, and SBP). Firstly, continuous variables, including age, BMI, and SBP, were converted into categorical variables based on clinical cutoff points (age: <30, ≥30 to <40, ≥40 to <50, ≥50 to <60, ≥60 to <70, ≥70 years old; BMI: <18.5, ≥18.5, <25, ≥25 kg/m^2^; SBP: <140, ≥140 mmHg) (29). Secondly, in addition to the stratification factor itself, we adjusted each stratification for all other factors (age, sex, BMI, SBP, DBP, ALT, AST, sCr, drinking status, family history of diabetes, and smoking status). Finally, the likelihood ratio test was used to determine the presence or absence of interaction terms in models with and without interaction terms.

We performed a series of sensitivity analyses to test the robustness of the results. We converted the TG/HDL-c ratio into a categorical variable according to the quartile and calculated the P-value for the trend to test the results of the TG/HDL-c ratio as a continuous variable and to explore the possibility of non-linearity. Previous studies have suggested that LDL-c, TC, and drinking status are significantly associated with diabetes (10, 25). Therefore, when exploring the association between TG/HDL-c ratio and incident diabetes in participants with prediabetes in other sensitivity analyses, we excluded participants with TC ≥5.0 mmol/l or LDL-c>2.5 mmol/l (11, 30). We also performed sensitivity analyses after excluding current and past drinkers. In addition, the proportion of missing data on drinking and smoking status was as high as 69.33%. Therefore, we further explored the association between the TG/HDL-c ratio and diabetes risk in patients with prediabetes when adjusted covariates did not include smoking and drinking status. Furthermore, we used a generalized additive model (GAM) to insert the continuity covariate into the equation as a curve to ensure the robustness of the results. We also explored the potential for unmeasured confounding between the TG/HDL-c ratio and diabetes risk by calculating E-values (31). All results were written according to the STROBE statement (32).

All analyses were conducted using the R statistical software packages (http://www.r-project.org, The R Foundation) and Empower Stats (X&Y Solutions, Inc., Boston, MA, http://www.empowerstats.com). Statistical significance was defined as P values less than 0.05 (two-sided).

## Results

### Characteristics of Participants

**Figure 2:**
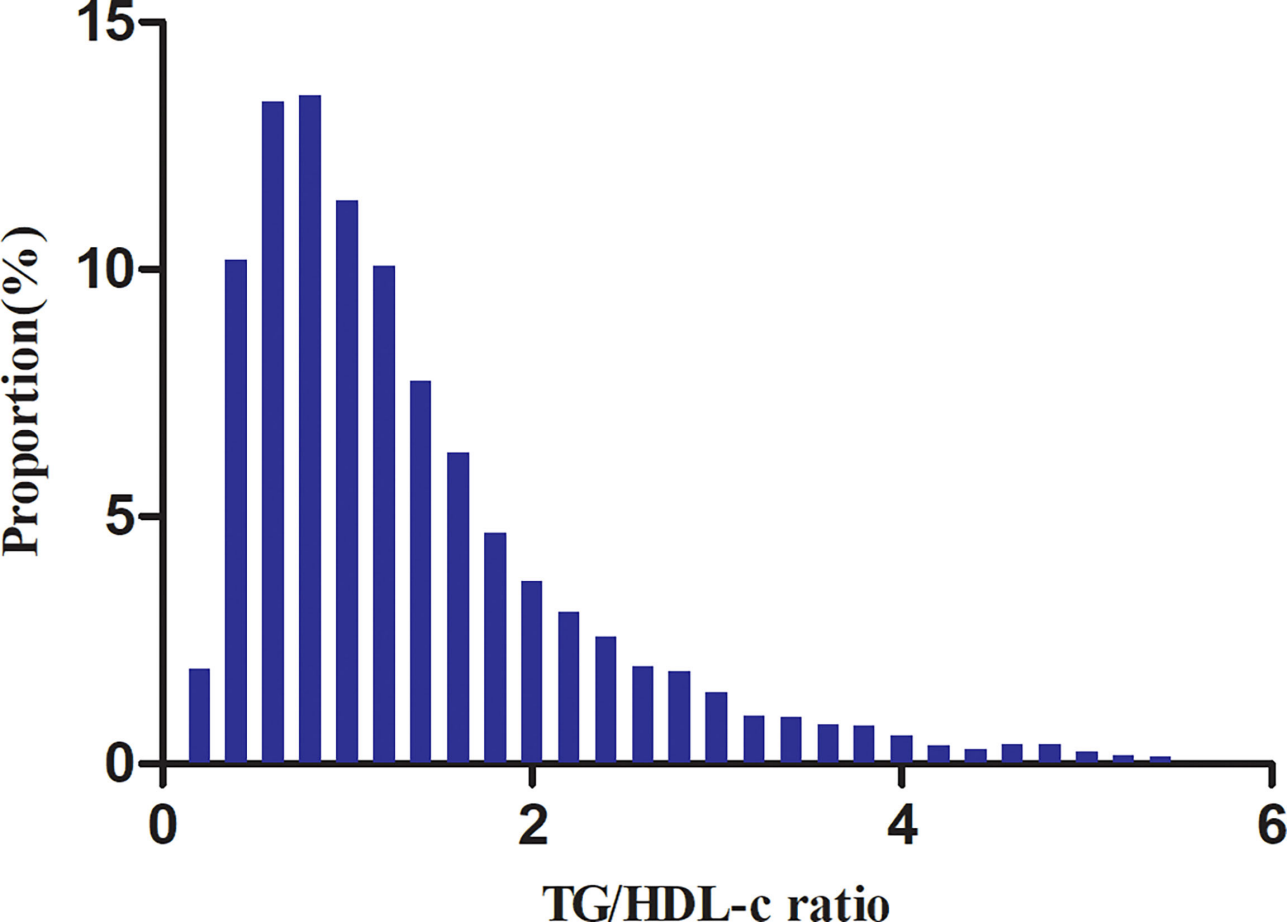
Distribution of the TG/HDL-c ratio. It presents a skewed distribution while being in the range from 0.039 to 5.453, with a median of 1.093.

**Table 1** shows the demographic and clinical characteristics of the study participants. The mean age was 50.95 ± 13.48 years old, and 9,745 (64.51%) were men. The median (interquartile range) TG/HDL-c ratio was 1.09 (0.69–1.72). The median follow-up time was 3.05 years, and 1,731 (11.46%) participants had a final diagnosis of diabetes. We assigned adults into subgroups using TG/HDL-c ratio quartiles (Q1: <0.692, Q2: 0.692–1.093, Q3: 11.092–1.718, Q4: ≥1.718). Compared with the lowest quartile (Q1: <0.692), age, height, weight, BMI, SBP, DBP, TC, TG, LDL-c, TG/HDL-c ratio, ALT, AST, BUN, and sCr increased significantly in the highest quartile (Q4: ≥1.718), whereas the opposite results were found in the HDL-c covariates. In addition, the proportion of men, current smokers, and current drinkers were higher in the highest quartile. The TG/HDL-c ratio presents a skewed distribution, ranging from 0.039 to 5.453, with a median of 1.093 (**Figure 2**).

**Table 1:** The baseline characteristics of participants.

TG/HDL ratio quartile	Q1 (<0.692)	Q2 (0.692–1.093)	Q3 (1.092–1.718)	Q4 (≥1.718)	P-value
Participants	3764	3,789	3,777	3,777	
Age (years)	48.68 ± 13.87	51.03 ± 13.96	52.11 ± 13.19	51.96 ± 12.60	<0.001
Sex					<0.001
Male	1,784 (47.40%)	2,368 (62.50%)	2,672 (70.74%)	2,921 (77.34%)	
Female	1,980 (52.60%)	1,421 (37.50%)	1,105 (29.26%)	856 (22.66%)	
Height (cm)	164.63 ± 8.21	166.25 ± 8.46	167.21 ± 8.35	168.32 ± 8.05	<0.001
Weight (kg)	62.44 ± 10.62	68.17 ± 11.17	71.43 ± 11.58	74.43 ± 11.79	<0.001
BMI (kg/m^2^)	22.96 ± 3.04	24.59 ± 3.13	25.47 ± 3.12	26.18 ± 3.07	<0.001
SBP (mmHg)	123.13 ± 17.59	127.36 ± 17.66	129.11 ± 17.37	130.13 ± 17.37	<0.001
DBP (mmHg)	75.25 ± 10.78	77.92 ± 11.11	79.64 ± 11.12	80.93 ± 10.90	<0.001
TC (mmol/L)	4.79 ± 0.89	4.98 ± 0.92	5.11 ± 0.93	5.24 ± 0.95	<0.001
TG (mmol/L)	0.76 ± 0.21	1.22 ± 0.24	1.74 ± 0.36	2.99 ± 1.05	<0.001
HDL-c (mmol/L)	1.56 ± 0.32	1.39 ± 0.23	1.28 ± 0.23	1.13 ± 0.24	<0.001
TG/HDL ratio	0.49 ± 0.13	0.88 ± 0.12	1.37 ± 0.18	2.67 ± 0.85	<0.001
LDL-c (mmol/L)	2.78 ± 0.67	2.96 ± 0.68	3.03 ± 0.70	3.01 ± 0.76	<0.001
ALT (U/L)	16.25 (12.10–23.00)	20.70 (15.00–29.20)	24.00 (17.20–35.00)	28.40 (20.00–41.60)	<0.001
AST (U/L)	23.33 ± 10.78	25.52 ± 11.62	26.90 ± 11.52	28.98 ± 12.25	<0.001
BUN (mmol/L)	4.98 ± 1.25	5.03 ± 1.26	5.03 ± 1.23	4.97 ± 1.21	0.049
Scr (μmol/L)	68.83 ± 15.44	72.73 ± 16.40	74.64 ± 15.78	75.63 ± 16.13	<0.001
Smoking status					<0.001
Current smoker	483 (12.83%)	746 (19.69%)	917 (24.28%)	1,120 (29.65%)	
Ever smoker	127 (3.37%)	146 (3.85%)	159 (4.21%)	171 (4.53%)	
Never smoker	3,154 (83.79%)	2,897 (76.46%)	2,701 (71.51%)	2,486 (65.82%)	
Drinking status					<0.001
Current drinker	77 (2.05%)	119 (3.14%)	159 (4.21%)	220 (5.82%)	
Ever drinker	471 (12.51%)	656 (17.31%)	696 (18.43%)	711 (18.82%)	
Never drinker	3,216 (85.44%)	3,014 (79.55%)	2,922 (77.36%)	2,846 (75.35%)	
Family history of diabetes					0.746
No	3,665 (97.37%)	3,699 (97.62%)	3,674 (97.27%)	3,674 (97.27%)	
Yes	99 (2.63%)	90 (2.38%)	103 (2.73%)	103 (2.73%)	

Continuous variables are summarized as mean (SD) or median (quartile interval); categorical variables are presented as percentages (%).

BMI, body mass index, TG, triglyceride; TC, total cholesterol; HDL-c, high-density lipoprotein cholesterol; LDL-c, low-density lipoprotein cholesterol; BUN, blood urea nitrogen; Scr, serum creatinine; ALT, alanine aminotransferase; AST, aspartate aminotransferase; DBP, diastolic blood pressure; SBP, systolic blood pressure; TG/HDL-C ratio, triglyceride-to-high-density lipoprotein cholesterol ratio.

### The Incidence Rate of Diabetes in Patients With Prediabetes

**Figure 3:**
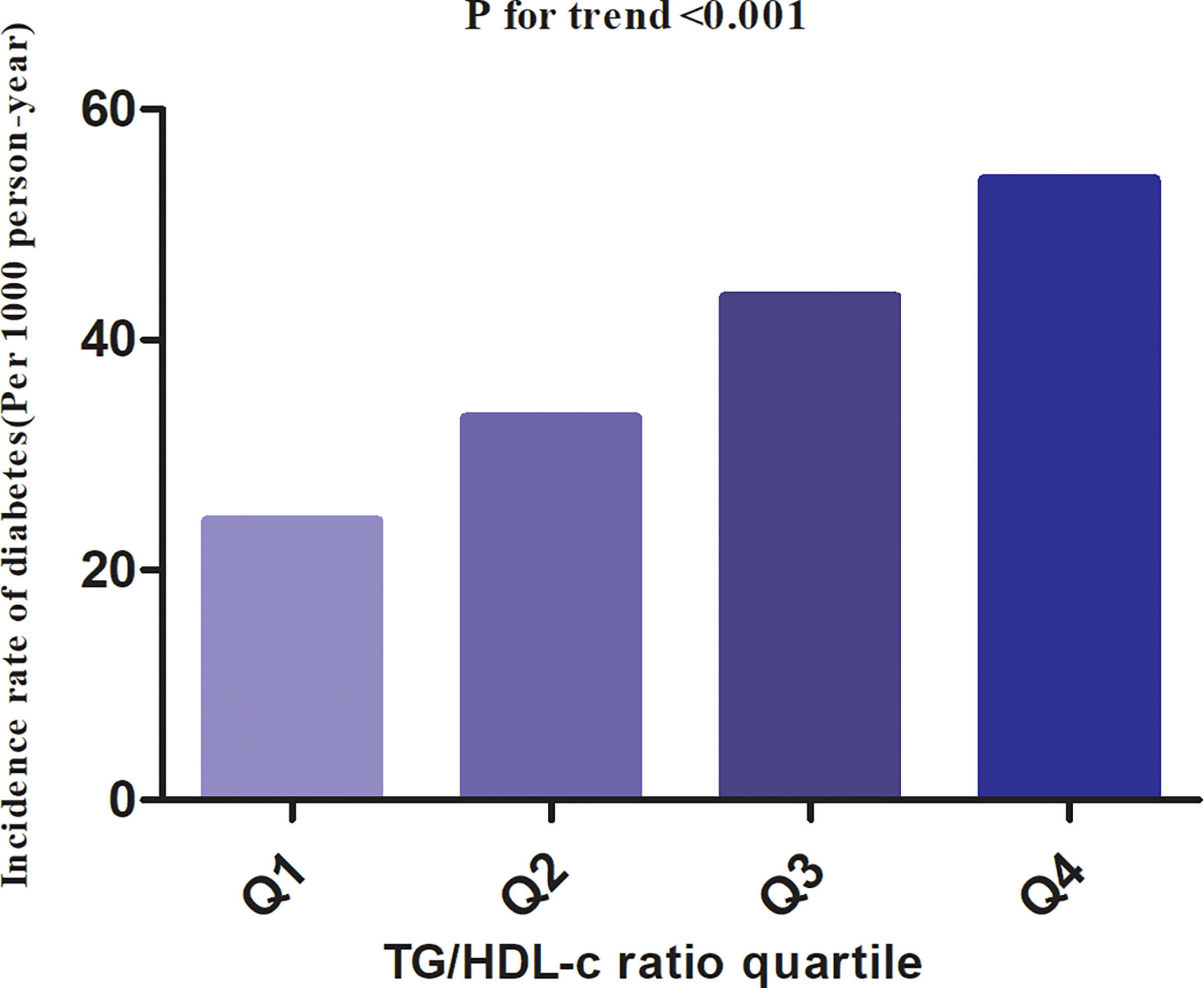
The incidence rate for diabetes(Per 1000 person-year) according to the quartiles of TG/HDL-c ratio. Participants with the highest TG/HDL-c ratio (Q4) had higher diabetes incidence rates than those with the lowest TG/HDL-c ratio (Q1) (P < 0.001 for trend).

**Figure 4:**
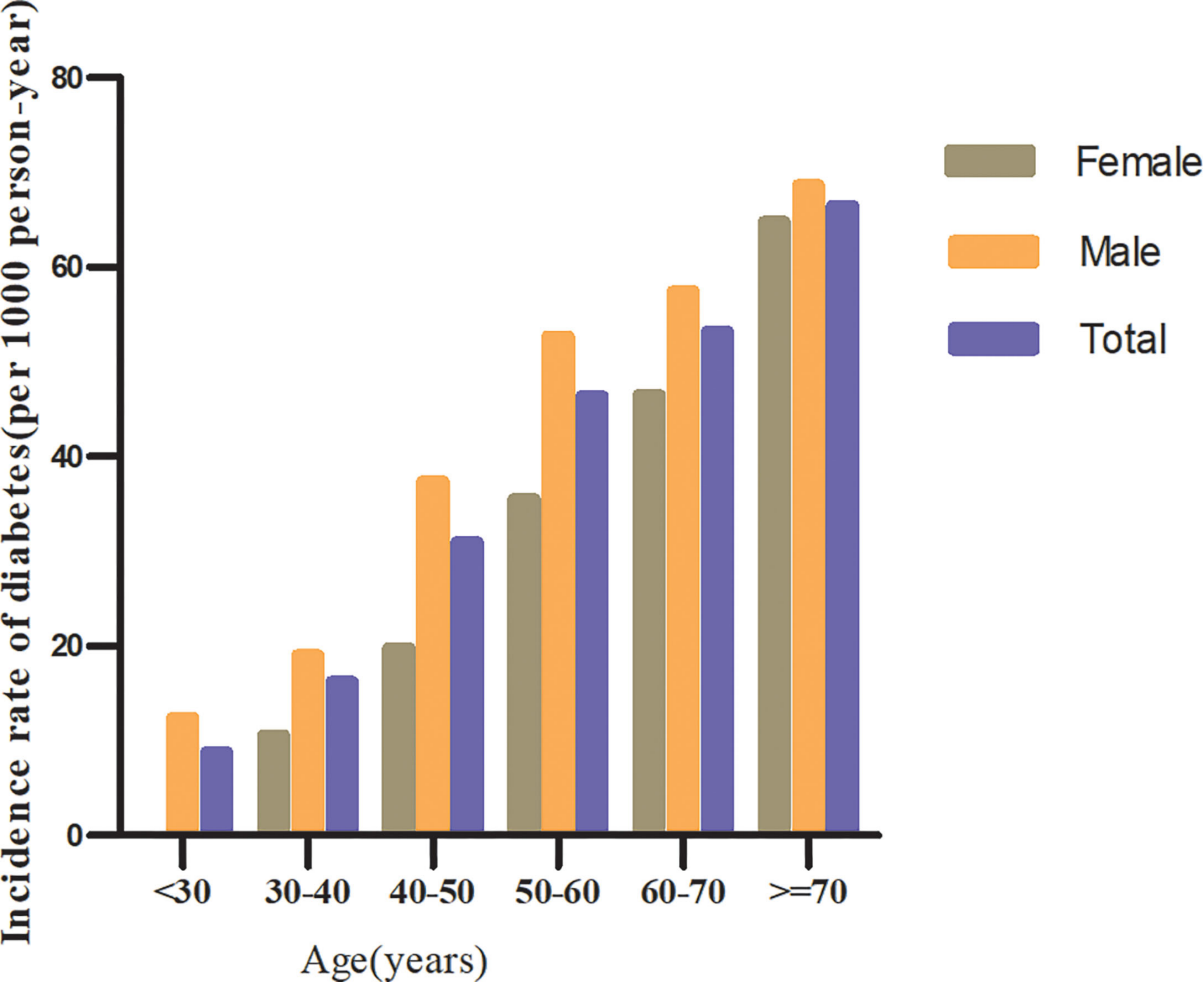
The incidence of diabetes (per 1,000 person-years) in prediabetic patients of age stratification by 10 intervals.

In individuals with prediabetes, 1,731 participants developed diabetes (38.93 per 1,000 person-years) during a median follow-up of 3.05 years. In particular, the incidence rate for diabetes among participants with prediabetes of the TG/HDL-c ratio quartiles was Q1: 24.5, Q2:33.20, Q3: 43.71, and Q4: 53.92 per 1,000 person-years, respectively. Participants with the highest TG/HDL-c ratio (Q4) had higher diabetes incidence rates than those with the lowest TG/HDL-c ratio (Q1) (P < 0.001 for trend) (**Table 2** and **Figure 3**). In the age stratification by 10 intervals, the incidence of diabetes in participants with prediabetes was higher in men than in women, regardless of their age group. It was also found that the incidence of diabetes increased with age in both men and women (**Figure 4**). The overall cumulative incidence of diabetes was 11.46% (1731/15017) over a median follow-up period of 3.05 years.

**Table 2:** The incidence rate of diabetes in participants with prediabetes (per 1,000 person-year).

TG/HDL-c ratio	Participants (n)	Diabetes events (n)	Incidence rate (per 1,000 person-year)
Total	15,107	1,731	38.93
Q1 (<0.692)	3,764	260	24.25
Q2 (0.692–1.093)	3,789	366	33.20
Q3 (1.092–1.718)	3,777	486	43.71
Q4 (≥1.718)	3,777	619	53.92
P for trend			<0.001

TG/HDL-C ratio, triglyceride-to-high-density lipoprotein cholesterol ratio; CI, confidence interval.

### Factors Influencing Risk of Diabetes in Patients With Prediabetes Analyzed by Univariate Cox Proportional Hazards Regression

Univariate analyses showed that diabetes in prediabetic patients was not associated with TC, LDL-c, BUN, and Scr (all P > 0.05) but was positively correlated with age, BMI, SBP, DBP, TG/HDL-c ratio, AST, ALT, current smoking, and family history of diabetes (all P < 0.05; **Table 3**).

**Table 3:** Factors influencing risk of diabetes in patients with prediabetes analyzed by univariate Cox proportional hazards regression.

	Statistics	HR (95% CI)	P-value
Age (years)	50.945 ± 13.482	1.028 (1.025, 1.032)	<0.00001
SEX			
Male	9,745 (64.507%)	Ref	
Female	5,362 (35.493%)	0.774 (0.697, 0.859)	<0.00001
BMI (kg/m^2^)	24.801 ± 3.315	1.098 (1.084, 1.112)	<0.00001
SBP (mmHg)	127.438 ± 17.697	1.014 (1.011, 1.016)	<0.00001
DBP (mmHg)	78.436 ± 11.179	1.015 (1.011, 1.019)	<0.00001
TC (mmol/L)	5.029 ± 0.936	1.011 (0.962, 1.062)	0.67121
TG/HDL-c ratio	1.353 ± 0.933	1.214 (1.164, 1.267)	<0.00001
TG (mmol/L)	1.678 ± 1.012	1.297 (1.248, 1.347)	<0.00001
HDL-c (mmol/L)	1.341 ± 0.300	1.326 (1.136, 1.547)	0.00035
LDL-c (mmol/L)	2.944 ± 0.712	1.007 (0.943, 1.076)	0.82528
ALT (U/L)	27.890 ± 23.002	1.005 (1.004, 1.006)	<0.00001
AST (U/L)	26.183 ± 11.736	1.010 (1.008, 1.012)	<0.00001
BUN (mmol/L)	5.003 ± 1.239	1.029 (0.991, 1.069)	0.13361
sCr (μmol/L)	72.961 ± 16.152	1.002 (0.999, 1.005)	0.25845
Smoking status			
Never smoker	11,238 (74.389%)	Ref	
Ever smoker	603 (3.992%)	1.507 (1.227, 1.853)	0.00010
Current smoker	3,266 (21.619%)	1.333 (1.198, 1.483)	<0.00001
Drinking status			
Never drinker	11,998 (79.420%)	Ref	
Ever drinker	2,534 (16.774%)	0.863 (0.758, 0.984)	0.02712
Current drinker	575 (3.806%)	1.156 (0.925, 1.445)	0.20104
Family history of diabetes			
NO	14,712 (97.385%)	Ref	
Yes	395 (2.615%)	1.325 (1.048, 1.675)	0.01863

Continuous variables are summarized as mean (SD) or median (quartile interval); categorical variables are presented as percentages (%).

TG, triglyceride; TC, total cholesterol; HDL-C, high-density lipoprotein cholesterol; LDL-C, low-density lipoprotein cholesterol; BUN, blood urea nitrogen; sCr serum creatinine; ALT, alanine aminotransferase; AST, aspartate aminotransferase; DBP, diastolic blood pressure; SBP, systolic blood pressure; TG/HDL-C ratio, triglyceride to high-density lipoprotein cholesterol ratio; HR, hazard ratios; CI, confidence; Ref, reference.

**Figure 5:**
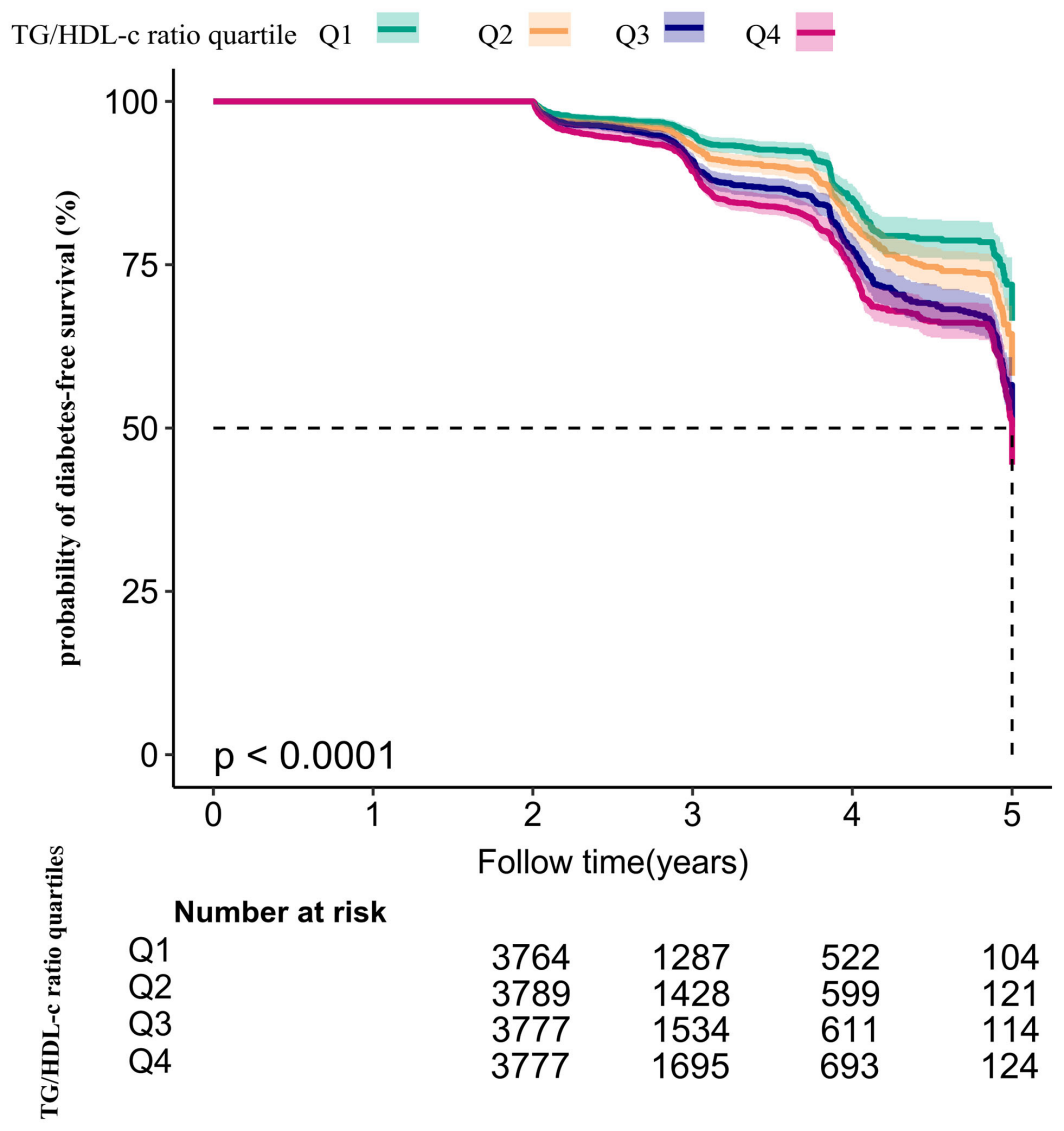
Kaplan–Meier event-free survival curve. Kaplan–Meier event-free survival curve. The probability of diabetes-free survival differed significantly between the TG/HDL-c ratio quartiles (log-rank test, P < 0.001). The probability of diabetes-free survival gradually increased with increasing TG/HDL-c ratio, suggesting that the group with the highest TG/HDL-c ratio had the highest risk of diabetes.

Kaplan–Meier survival curves for diabetes-free survival probability stratified by TG/HDL-c ratio quartile are shown in **Figure 5**. There were significant differences in the probability of diabetes-free survival between the TG/HDL-c ratio quartiles (log-rank test, P < 0.001). The probability of diabetes-free survival gradually decreased with increasing TG/HDL-c ratio, indicating that the group with the highest TG/HDL-c ratio had the highest risk of diabetes among patients with prediabetes.

### The Results of Multivariable Analyses Using Cox Proportional-Hazards Regression Models

Three models were constructed using the Cox proportional-hazards regression model to investigate the relationship between the TG/HDL-c ratio and incident diabetes in participants with prediabetes (**Table 4**). In the unadjusted model (crude model), an increase of 1 unit of the TG/HDL-c ratio was linked to a 21.4% increase in the risk of diabetes (HR = 1.214, 95% CI: 1.164–1.267, P < 0.001). In the minimally adjusted model (model I), each additional 1 unit of the TG/HDL-c ratio increased the risk of diabetes by 13% in prediabetic participants (HR = 1.130, 95% CI 1.080–1.183, P < 0.001). In the fully adjusted model (model II), each additional 1 unit of the TG/HDL-c ratio was accompanied by an 11.1% increase in diabetes risk in patients with prediabetes (HR = 1.111, 95% CI 1.061–1.164). The distribution of the confidence intervals indicated that the relationship between the TG/HDL-c ratio and diabetes risk in participants with diabetes obtained by the model was reliable.

**Table 4:** Relationship between TG/HDL-c ratio and the risk of diabetes in prediabetic patients in different models.

Exposure	Crude model (HR, 95% CI)	Model I (HR, 95% CI) P	Model II (HR, 95% CI) P	Model III (HR, 95% CI) P
TG/HDL-c ratio	1.214 (1.164, 1.267) <0.001	1.130 (1.080, 1.183) <0.001	1.111 (1.061, 1.164) <0.001	1.106 (1.056, 1.158) <0.001
TG/HDL-c ratio quartile				
Q1	Ref	Ref	Ref	Ref
Q2	1.295 (1.105, 1.518) 0.001	1.095 (0.933, 1.286) 0.268	1.096 (0.933, 1.288) 0.263	1.105 (0.939, 1.299) 0.229
Q3	1.675 (1.440, 1.947) <0.001	1.291 (1.106, 1.508) 0.001	1.252 (1.072, 1.462) 0.005	1.288 (1.100, 1.508) 0.002
Q4	1.944 (1.681, 2.247) <0.001	1.457 (1.252, 1.696) <0.001	1.397 (1.200, 1.627) <0.001	1.415 (1.212, 1.653) 0.001
P for trend	<0.001	<0.001	<0.001	<0.001

Crude model: we did not adjust for other covariates.

Model I: We adjusted for age, sex, BMI.

Model II: We adjusted for age, sex, BMI, SBP, DBP, ALT, AST, sCr, smoking status, drinking status, and a family history of diabetes.

Model III: We adjusted for age (smooth), sex, BMI (smooth), SBP (smooth), DBP (smooth), ALT (smooth), AST (smooth), sCr (smooth), smoking status, drinking status, and family history of diabetes.

HR, hazard ratios; CI, confidence; Ref, reference.

### Sensitivity Analysis

A series of sensitivity analyses were performed to verify the robustness of our findings. We first transformed the TG/HDL-c ratio from a continuous variable to a categorical variable (according to quartiles) and then returned the categorically changed TG/HDL-c ratio to the regression equation. The results showed that the trends in effect sizes (HR) between groups were equidistant after transforming the TG/HDL-c ratio into a categorical variable. The P for the trend was consistent with the result when the TG/HDL-c ratio was a continuous variable (**Table 4**).

Additionally, we used a GAM to introduce the continuity covariate as a curve into the equation. Model III’s outcome in **Table 4** demonstrated that this remained reasonably consistent with the fully corrected model (HR = 1.106, 95% CI: 1.056–1.158, P < 0.001). In addition, we generated an E-value to assess sensitivity to unmeasured confounding factors. The E-value (1.46) was more significant than the relative risk (1.33) of unmeasured confounders and the TG/HDL-c ratio, suggesting that unmeasured or unknown confounders had little effect on the relationship between the TG/HDL-c ratio and incident diabetes in patients with prediabetes.

Furthermore, dyslipidemia was significantly associated with diabetes. We excluded participants with LDL-c >2.5 mmol/l in the sensitivity analyses. After correcting for confounding variables (including age, sex, BMI, SBP, DBP, ALT, AST, sCr, smoking status, drinking status, and family history of diabetes), the findings indicated that the TG/HDL-c ratio was also positively associated with diabetes risk (HR = 1.109, 95% CI: 1.034–1.190, P = 0.004) (**Table 5**). We also excluded participants with TC ≥5.0 mmol/l for the sensitivity analyses. After correcting for confounding variables (including age, sex, BMI, SBP, DBP, ALT, AST, sCr, smoking status, drinking status, and family history of diabetes), the results suggested that the TG/HDL-c ratio was still positively associated with incident diabetes in participants with diabetes (HR = 1.103, 95% CI: 1.020–1.193, P = 0.014). In addition, restricting the analysis to participants who never drank alcohol (adjusted for age, sex, BMI, SBP, DBP, ALT, AST, sCr, smoking status, and family history of diabetes), the results suggested that the HR between the TG/HDL-c ratio and the risk of diabetes in prediabetic patients was 1.115 (95% CI : 1.059–1.175, P < 0.001). Further, we explored the relationship between the TG/HDL-c ratio and the risk of progression from prediabetes to diabetes (**Table 5** model IV) after excluding smoking and drinking status. The results suggested that the HR between the TG/HDL-c ratio and the risk of diabetes in patients with prediabetes was 1.121 (95% CI: 1.045–1.202, P = 0.001) (**Table 5**). The results obtained from all the sensitivity analyses indicated the robustness of our findings.

**Table 5:** Relationship between TG/HDL-c ratio and the risk of diabetes in participants with prediabetes in different sensitivity analyses.

Exposure	Model I (HR, 95% CI) P	Model I (HR, 95% CI) P	Model III (HR, 95%CI) P	Model IV (HR, 95% CI) P
TG/HDL-c ratio	1.109 (1.034, 1.190) 0.004	1.103 (1.020, 1.193) 0.014	1.115 (1.059, 1.175) <0.001	1.121 (1.045, 1.202) 0.001
**T**G/HDL-c ratio quartile				
** Q1**	Ref	Ref	Ref	Ref
** Q2**	1.123 (0.900, 1.401) 0.305	1.306 (0.976, 1.749) 0.073	1.133 (0.948, 1.354) 0.169	1.128 (0.905, 1.407) 0.284
** Q3**	1.445 (1.166, 1.792) <0.001	1.602 (1.200, 2.138) 0.001	1.296 (1.091, 1.539) 0.003	1.461 (1.179, 1.810) <0.001
** Q4**	1.478 (1.191, 1.833) <0.001	1.691 (1.280, 2.233) <0.001	1.404 (1.184, 1.666) <0.001	1.505 (1.214, 1.866) <0.001
**P for trend**	<0.001	<0.001	<0.001	<0.001

Model I was a sensitivity analysis in participants without TC ≥5.0 mmol/L (N = 7738). We adjusted for age, sex, BMI, SBP, DBP, ALT, AST, sCr, smoking status, drinking status, and a family history of diabetes.

Model II was a sensitivity analysis in participants without LDL-c >2.5 mmol/L (N = 4218). We adjusted for age, sex, BMI, SBP, DBP, ALT, AST, sCr, smoking status, drinking status, and a family history of diabetes.

Model III was a sensitivity analysis of participants who had never consumed alcohol (N = 11998). We adjusted for age, sex, BMI, SBP, DBP, ALT, AST, sCr, smoking status, and a family history of diabetes.

Model IV was a sensitivity analysis of all participants with prediabetes (N = 15,107). We adjusted for age, sex, BMI, SBP, DBP, ALT, AST, sCr, and family history of diabetes.

### Cox Proportional Hazards Regression Model With Cubic Spline Functions to Address Non-Linearity

**Figure 6:**
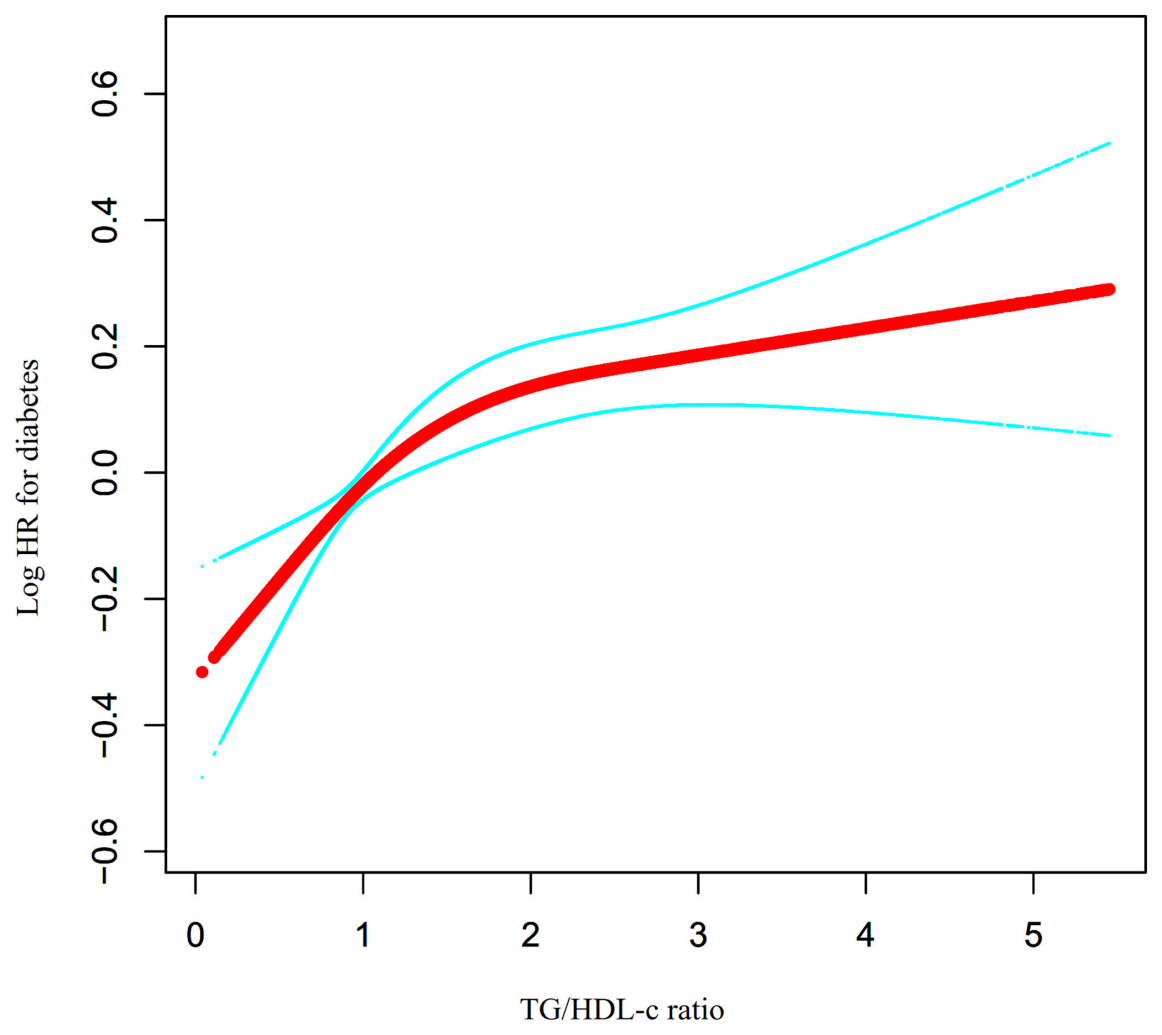
The non-linear relationship between TG/HDL-c ratio and the risk of diabetes in prediabetic participants. We used a Cox proportional hazards regression model with cubic spline functions to evaluate the relationship between TG/HDL-c ratio and diabetes risk. The result showed that the relationship between the TG/HDL-c ratio and diabetes risk in prediabetic patients was non-linear, with the inflection point of TG/HDL-c ratio being 1.415.

Through the Cox proportional hazards regression model with cubic spline functions, we noticed that the link between the TG/HDL-c ratio and incident diabetes in prediabetic patients was likewise non-linear (**Figure 6**). Therefore, data were fitted to a two-piecewise Cox proportional hazards regression model to fit two different slopes. We also fitted the data using a standard binary two-piecewise Cox proportional-hazards regression model based on the sensitivity analysis and selected the best-fit model through the log-likelihood ratio test (**Table 6**). The P-value for the log-likelihood ratio test was 0.021. Using a recursive algorithm, we first determined that the inflection point of the TG/HDL-c ratio was 1.415 and then calculated the HR and CI on the left and right of the inflection point using a two-piecewise Cox proportional hazards regression model. The HR was 1.336 on the left side of the inflection point (95% CI: 1.134–1.573). The HR was 1.055 on the right side of the inflection point (95% CI: 0.988–1.126), but the difference was not statistically significant.

**Table 6:** The result of the two-piecewise linear regression model.

Incident prediabetes	HR, 95%CI	P
Fitting model by standard Cox regression	1.111 (1.061, 1.164)	<0.001
Fitting model by two-piecewise Cox regression		
Inflection points of TG/HDL-c ratio	1.415	
** ≤1.415**	1.336 (1.134, 1.573)	<0.001
** >1.415**	1.055 (0.988, 1.126)	0.108
P for log-likelihood ratio test	0.021	

We adjusted for age, sex, BMI, SBP, DBP, ALT, AST, sCr, smoking status, drinking status, and a family history of diabetes.

### The Results of Subgroup Analyses

In all the prespecified or exploratory subgroups evaluated (**Table 7**), there was no significant interaction in age, BMI, drinking status, smoking status, and SBP. In contrast, significant interactions were detected in sex. Specifically, a stronger association between the TG/HDL-c ratio and the risk of incident diabetes was observed in female participants (HR = 1.210, 95% CI: 1.100–1.331, P < 0.001). In contrast, a weaker relationship between the TG/HDL-c ratio and diabetes risk was observed in male participants with prediabetes (HR = 1.084, 95% CI: 1.029–1.142, P = 0.003). However, considering that drinking and smoking may be related to sex, the proportion of missing data for drinking and smoking status was 69.33%. This may be the reason for the sex differences in the subgroup analysis. Therefore, we performed a series of subgroup analyses, adjusted for covariates excluding smoking and drinking status (**Supplementary Table 1**). The results suggest that sex did not affect the relationship between the TG/HDL-c ratio and the risk of diabetes in patients with prediabetes.

**Table 7:** Stratified associations between TG/HDL-c ratio and diabetes in participants with prediabetes by age, sex, BMI, SBP, smoking status, and drinking status.

Characteristic	No. of participants	HR (95% CI) P value	P for interaction
Age, years			0.105
<30	589	1.175 (0.627, 2.201) 0.616	
30 to <40	3,047	1.430 (1.251, 1.636) <0.001	
40 to <50	3,387	1.232 (1.122, 1.353) <0.001	
50 to <60	3,818	1.034 (0.953, 1.122) 0.424	
60 to <70	2,916	1.036 (0.946, 1.133) 0.447	
≥70	1,350	1.032 (0.898, 1.185) 0.660	
Sex			0.045
Male	9,745	1.085 (1.030, 1.143) 0.002	
Female	5,362	1.214 (1.104, 1.335) <0.001	
BMI (kg/m^2^)			0.523
<18.5	257	1.273 (0.324, 5.012) 0.730	
≥18.5, <25	7,881	1.181 (1.093, 1.275) <0.001	
≥25	6,969	1.096 (1.036, 1.159) 0.001	
Drinking status			0.785
Current	575	1.172 (0.963, 1.425) 0.113	
Ever	2,534	1.084 (0.970, 1.210) 0.154	
Never	11,998	1.114 (1.057, 1.173) <0.001	
Smoking status			0.177
Current	3266	1.064 (0.981, 1.154) 0.134	
Ever	603	1.077 (0.918, 1.263) 0.362	
Never	11,238	1.143 (1.078, 1.211) <0.001	
SBP (mmHg)			0.716
<140	11,735	1.110 (1.050, 1.173) <0.001	
≥140	3,372	1.095 (1.012, 1.185) 0.024	

Model adjusted for age, sex, BMI, SBP, DBP, ALT, AST, sCr, smoking status, drinking status, and family history of diabetes.

In each case, the model is not adjusted for the stratification variable.

HR, hazard ratios; CI, confidence; CE: Please ensure that all reference citations are styled correctly., reference.

## Discussion

This retrospective cohort study was designed to examine the link between the TG/HDL-C ratio and incident diabetes in prediabetic patients. We found that an increase in the TG/HDL-c ratio was related to a significantly increased risk of diabetes in prediabetic patients. In addition, a threshold effect curve was also found, and different relationships between the TG/HDL-c ratio and the risk of diabetes were detected on both sides of the inflection point.

The actual 5-year rate of progression from prediabetes to diabetes in a Japanese study was 8.5% (33). The 7-year rate of progression to diabetes in American patients with prediabetes aged 70–79 years is 10.6% (34). In the present study, the cumulative incidence of diabetes in participants with prediabetes was 11.46% over a median follow-up time of 3.05 years. These differences in the incidence of diabetes among these patients may be due to differences in the participants’ age and ethnicity. It is worth noting that all studies have confirmed that patients with prediabetes have a high risk of developing diabetes. Therefore, it is essential to actively search for various other risk factors for the progression from prediabetes to diabetes.

The TG/HDL-c ratio has often been used to evaluate insulin resistance in previous studies (35, 36). Recent studies have shown that an elevated TG/HDL-c ratio is associated with the risk of incident diabetes (15, 17, 37). A retrospective cohort study enrolled 114,787 participants and found that after adjusting for potential confounding factors, an increase of 1 unit of TG/HDL-c ratio was linked with a 15.9% increase in the risk of diabetes (HR = 1.159, 95% CI: 1.104–1.215) (17). Another study included 11,946 participants without baseline diabetes from a rural Chinese cohort. The results showed that people in the highest TG/HDL-c ratio quartile had a higher diabetes risk than those in the lowest quartile (HR: 2.11, 95% CI: 1.55–2.86) (38). Our findings are consistent with previous results showing that the TG/HDL-c ratio is positively related to the incidence of diabetes. However, to the best of our knowledge, the present study is the first to reveal a relationship between the TG/HDL-c ratio and the risk of progression from prediabetes to diabetes. Meanwhile, the sensitivity analysis found that this relationship still exists among participants with TC < 5.0 mmol/l, LDL-c ≤ 2.5 mmol/l, or those who never engaged in drinking. The results mentioned above have confirmed the relationship’s stability between TG/HDL-c ratio and diabetes risk in patients with prediabetes. These results provide a reference for optimizing interventions to reduce the risk of developing diabetes in patients with prediabetes.

Insulin resistance may be the main mechanism by which high TG/HDL-c ratios are associated with a higher incidence of diabetes in prediabetic patients (36). Additionally, pancreatic β-cell dysfunction may be one of the possible explanations for the relationship between the TG/HDL-c ratio and the progression of prediabetes (15, 39). Lower levels of HDL-c reduce cholesterol efflux, leading to cholesterol accumulation in the pancreatic β-cells. This leads to β-cell dysfunction, including elevated blood glucose, impaired insulin secretion, and β-cell apoptosis (40, 41). Furthermore, elevated TG levels elevate nitric oxide and ceramide levels, which induce β-cell apoptosis and impair glucose-stimulated insulin secretion (15, 42). These mechanisms may reflect the relationship between the TG/HDL-c ratio and the incidence of diabetes.

Furthermore, the present study observed a non-linear relationship between the TG/HDL-c ratio and the risk of diabetes in patients with prediabetes for the first time. This study used a two-piecewise Cox proportional hazards regression model to clarify the non-linear relationships. The results showed a non-linear relationship and saturation effect between the TG/HDL-c ratio and the risk of diabetes in patients with prediabetes. The inflection point of the TG/HDL-c ratio was 1.415 after adjusting for confounders. When the TG/HDL-c ratio was below 1.415, a 1-unit increase in the TG/HDL-c ratio was associated with a 33.6% increase in the incidence rate of diabetes. The risk of diabetes did not increase with an increase in the TG/HDL-c ratio when the TG/HDL-c ratio was greater than 1.415.

It could be found that compared to participants with a TG/HDL-c ratio >1.415, participants with TG/HDL-c ratio ≤1.415 were generally younger and had lower BMI, SBP, DBP, LDL-c, TC, AST, and ALT. In addition, those with a TG/HDL-c ratio ≤1.415 had a lower proportion of currently drinking (**Supplementary Table 2**). However, these indicators are closely related to diabetes (21, 43–46). When the TG/HDL-c ratio was higher than 1.415, the TG/HDL-c ratio had a relatively weak effect on diabetes owing to the presence of these risk factors. On the contrary, when the TG/HDL-c ratio was less than 1.415, the level of these risk factors for diabetes was lower, the impact on diabetes was reduced, and the effect of the TG/HDL-c ratio was relatively enhanced. The discovery of a curvilinear relationship between the TG/HDL-c ratio and diabetes in prediabetic patients has excellent clinical value. It provides a reference for optimizing diabetes prevention decision-making and promoting clinical consultation in patients with prediabetes.

Our study had several strengths: (i) The total sample size was relatively large. (ii) To the best of our knowledge, this is the first time that Chinese prediabetic people have been used as a research population to explore the association between TG/HDL-c ratio and the risk of diabetes. (iii) Compared to previous research, research addressing non-linearity is a significant improvement. (iv) Multiple imputations were employed to handle missing data. This method can maximize the statistical power and minimize the potential bias caused by missing covariate information. (v) In this study, we tested the robustness of the results through a series of sensitivity analyses (conversion of target-independent variable form, subgroup analysis, using a GAM to insert the continuity covariate into the equation as a curve, calculating E-values to explore the potential for unmeasured confounding, and reanalyzing the relationship between the TG/HDL-c ratio and the risk of diabetes in prediabetic patients with TC <5.0 mmol/l, LDL-c ≤2.5 mmol/l, or never drinking) to ensure the reliability of the results.

The potential limitations of this study are as follows. First, since all participants were of Chinese descent, further research is needed to determine the relationship between the TG/HDL-c ratio and the risk of diabetes in prediabetic individuals with different genetic backgrounds. Second, diabetes was defined as a fasting plasma glucose (FPG) ≥7.00 mmol/l and/or self-reported diabetes during the follow-up period, but not a 2-h oral glucose tolerance test or measurement of glycosylated hemoglobin level, which may underestimate the incidence of diabetes. Additionally, the type of diabetes was not determined. Type 2 diabetes mellitus (T2DM) is the most common type of diabetes, accounting for more than 90% of all diabetes cases in China (47). Therefore, our findings are representative of T2DM patients. Third, this study is based on a secondary analysis of published data; therefore, it is impossible to adjust variables not included in the original dataset, such as insulin concentration, TNF, and IL-6. However, we calculated the E-value to quantify the potential impact of unmeasured confounders and found that unmeasured confounders were unlikely to explain the results. Likewise, detailed measures of additional parameters and methods of assessing information cannot be specified. Fourth, for such a large sample of participants, the median follow-up time was 3.05 years, the longest follow-up time was 5 years, and 1,350 participants (8.94%) were older than 70 years old. Potential censoring by death is unavoidable. Therefore, if the original research data contained information on the censoring by death, then the competitive risk model would be a more appropriate choice. In the future, we can consider designing our studies and collect the potential censoring by death. We could analyze the relationship between the TG/HDL-c ratio and diabetes in patients with prediabetes through a competitive risk model. In addition, this retrospective observational study provided an association inference rather than establishing a causal relationship between the TG/HDL-c ratio and the risk of diabetes in prediabetic patients. Finally, the present study only measured TG, HDL-c, and other parameters at baseline and did not consider the TG/HDL-c ratio changes over time. In the future, we can also consider designing our studies or collaborating with other researchers to collect as many variables as possible, including information on the evolution of TG/HDL-C during patient follow-up.

## Conclusion

This study demonstrates a positive and non-linear relationship between the TG/HDL-c ratio and incident diabetes in Chinese adults with prediabetes. There is a saturation effect between the TG/HDL-c ratio and the risk of diabetes in patients with prediabetes. When the TG/HDL-c ratio was <1.415, there was a significant positive association with the risk of progression from prediabetes to diabetes. This result is expected to provide a reference for clinicians to control dyslipidemia in prediabetic patients. It makes sense to lower the TG/HDL-c ratio below the inflection point by aggressive intervention to lower TG or increase HDL-c levels from a treatment perspective. Reducing the TG/HDL-c ratio can significantly reduce the risk of progression from diabetes to diabetes when the TG/HDL-c ratio is below the inflection point (1.415).

## Data Availability Statement

The datasets presented in this study can be found in online repositories. The names of the repository/repositories and accession number(s) can be found in the article/**Supplementary Material**.

## Ethics Statement

The studies involving human participants were reviewed and approved by the Rich Healthcare Group Review Board. Written informed consent for participation was not required for this study in accordance with the national legislation and the institutional requirements.

## Author Contributions

YS, ZW, and ZH conceived the research, drafted the manuscript, and performed the statistical analysis. HH and YH revised the manuscript and designed the study. All authors have read and approved the final manuscript.

## Conflict of Interest

The authors declare that the research was conducted in the absence of any commercial or financial relationships that could be construed as a potential conflict of interest.

## Publisher’s Note

All claims expressed in this article are solely those of the authors and do not necessarily represent those of their affiliated organizations, or those of the publisher, the editors and the reviewers. Any product that may be evaluated in this article, or claim that may be made by its manufacturer, is not guaranteed or endorsed by the publisher.

## Abbreviations

TG/HDL-c, triglyceride-to-HDL cholesterol ratio; BMI, body mass index; TG, triglyceride; TC, total cholesterol; HDL-c, high-density lipoprotein cholesterol; LDL-c, low-density lipoprotein cholesterol; BUN, blood urea nitrogen; sCr, serum creatinine; ALT, alanine aminotransferase; AST, aspartate aminotransferase; DBP, diastolic blood pressure; SBP, systolic blood pressure; T2DM, type 2 diabetes mellitus; FPG, fasting plasma glucose; IDF, International Diabetes Federation; GAM, generalized additive model; HR, hazard ratio; CI, confidence interval; Ref, reference.

## References

[1] Sun H Saeedi P Karuranga S Pinkepank M Ogurtsova K Duncan BB. IDF Diabetes Atlas: Global, Regional and Country-Level Diabetes Prevalence Estimates for 2021 and Projections for 2045. Diabetes Res Clin Pract (2022) 183:109119. doi: 10.1016/j.diabres.2021.109119 34879977 PMC11057359

[2] Cho NH Shaw JE Karuranga S Huang Y Da RFJ Ohlrogge AW. IDF Diabetes Atlas: Global Estimates of Diabetes Prevalence for 2017 and Projections for 2045. Diabetes Res Clin Pract (2018) 138:271–81. doi: 10.1016/j.diabres.2018.02.023 29496507

[3] Punthakee Z Goldenberg R Katz P. Definition, Classification and Diagnosis of Diabetes, Prediabetes and Metabolic Syndrome. Can J Diabetes (2018) 42 Suppl:1, S10–S15. doi: 10.1016/j.jcjd.2017.10.003 29650080

[4] Nathan DM. The Diabetes Control and Complications Trial/Epidemiology of Diabetes Interventions and Complications Study at 30 Years: Overview. Diabetes Care (2014) 37(1):9–16. doi: 10.2337/dc13-2112 24356592 PMC3867999

[5] Peters SA Huxley RR Woodward M. Diabetes as Risk Factor for Incident Coronary Heart Disease in Women Compared With Men: A Systematic Review and Meta-Analysis of 64 Cohorts Including 858,507 Individuals and 28,203 Coronary Events. Diabetologia (2014) 57(8):1542–51. doi: 10.1007/s00125-014-3260-6 24859435

[6] Saeedi P Petersohn I Salpea P Malanda B Karuranga S Unwin N. Williams: Global and Regional Diabetes Prevalence Estimates for 2019 and Projections for 2030 and 2045: Results From the International Diabetes Federation Diabetes Atlas, 9(Th) Edition. Diabetes Res Clin Pract (2019) 157:107843. doi: 10.1016/j.diabres.2019.107843 31518657

[7] Wang L Gao P Zhang M Huang Z Zhang D Deng Q. Wang: Prevalence and Ethnic Pattern of Diabetes and Prediabetes in China in 2013. JAMA (2017) 317(24):2515–23. doi: 10.1001/jama.2017.7596 PMC5815077 28655017

[8] Tabák AG Herder C Rathmann W Brunner EJ Kivimäki M. Prediabetes: A High-Risk State for Diabetes Development. Lancet (2012) 379(9833):2279–90. doi: 10.1016/S0140-6736(12)60283-9 PMC3891203 22683128

[9] Schofield JD Liu Y Rao-Balakrishna P Malik RA Soran H. Diabetes Dyslipidemia. Diabetes Ther (2016) 7(2):203–19. doi: 10.1007/s13300-016-0167-x PMC4900977 27056202

[10] Mooradian AD. Dyslipidemia in Type 2 Diabetes Mellitus. Nat Clin Pract Endocrinol Metab (2009) 5(3):150–9. doi: 10.1038/ncpendmet1066 19229235

[11] Turak O Afşar B Ozcan F Öksüz F Mendi MA Yayla Ç. The Role of Plasma Triglyceride/High-Density Lipoprotein Cholesterol Ratio to Predict New Cardiovascular Events in Essential Hypertensive Patients. J Clin Hypertens (Greenwich) (2016) 18(8):772–7. doi: 10.1111/jch.12758 PMC8031528 26694089

[12] Fukuda Y Hashimoto Y Hamaguchi M Fukuda T Nakamura N Ohbora A. Triglycerides to High-Density Lipoprotein Cholesterol Ratio is an Independent Predictor of Incident Fatty Liver; a Population-Based Cohort Study. Liver Int (2016) 36(5):713–20. doi: 10.1111/liv.12977 26444696

[13] Lin D Qi Y Huang C Wu M Wang C Li F. Associations of Lipid Parameters With Insulin Resistance and Diabetes: A Population-Based Study. Clin Nutr (2018) 37(4):1423–9. doi: 10.1016/j.clnu.2017.06.018 28673690

[14] Young KA Maturu A Lorenzo C Langefeld CD Wagenknecht LE Chen YI. The Triglyceride to High-Density Lipoprotein Cholesterol (TG/HDL-C) Ratio as a Predictor of Insulin Resistance, β-Cell Function, and Diabetes in Hispanics and African Americans. J Diabetes Complications (2019) 33(2):118–22. doi: 10.1016/j.jdiacomp.2018.10.018 PMC6331279 30522790

[15] Lim TK Lee HS Lee YJ. Triglyceride to HDL-Cholesterol Ratio and the Incidence Risk of Type 2 Diabetes In Community Dwelling Adults: A Longitudinal 12-Year Analysis of the Korean Genome and Epidemiology Study. Diabetes Res Clin Pract (2020) 163:108150. doi: 10.1016/j.diabres.2020.108150 32305400

[16] Gong R Liu Y Luo G Liu W Jin Z Xu Z. Associations of TG/HDL Ratio With the Risk of Prediabetes and Diabetes in Chinese Adults: A Chinese Population Cohort Study Based on Open Data. Int J Endocrinol (2021) 2021:9949579. doi: 10.1155/2021/9949579 34306073 PMC8282372

[17] Chen Z Hu H Chen M Luo X Yao W Liang Q. Association of Triglyceride to High-Density Lipoprotein Cholesterol Ratio and Incident of Diabetes Mellitus: A Secondary Retrospective Analysis Based on a Chinese Cohort Study. Lipids Health Dis (2020) 19(1):33. doi: 10.1186/s12944-020-01213-x 32131838 PMC7057518

[18] Chen Y Zhang XP Yuan J Cai B Wang XL Wu XL. Association of Body Mass Index and Age With Incident Diabetes in Chinese Adults: A Population-Based Cohort Study. BMJ Open (2018) 8(9):e021768. doi: 10.1136/bmjopen-2018-021768 PMC6169758 30269064

[19] 2. Classification and Diagnosis of Diabetes: Standards of Medical Care in Diabetes-2021. Diabetes Care (2021) 44(Suppl 1):S15–33. doi: 10.2337/dc21-S002 34135016

[20] Zhang N Hu X Zhang Q Bai P Cai M Zeng TS. Non-High-Density Lipoprotein Cholesterol: High-Density Lipoprotein Cholesterol Ratio is an Independent Risk Factor for Diabetes Mellitus: Results From a Population-Based Cohort Study. J Diabetes (2018) 10(9):708–14. doi: 10.1111/1753-0407.12650 29437292

[21] Niu H Zhou Y. Nonlinear Relationship Between AST-To-ALT Ratio and the Incidence of Type 2 Diabetes Mellitus: A Follow-Up Study. Int J Gen Med (2021) 14:8373–82. doi: 10.2147/IJGM.S341790 PMC8608244 34819745

[22] Choukem SP Dimala CA. BMI and Diabetes Risk in Low-Income and Middle-Income Countries. Lancet (2021) 398(10296):190–2. doi: 10.1016/S0140-6736(21)01425-2 34274054

[23] Liu Y Zhou L Liu Z Ma Y Lin L Zhu Y. Higher Blood Urea Nitrogen and Urinary Calcium: New Risk Factors for Diabetes Mellitus in Primary Aldosteronism Patients. Front Endocrinol (Lausanne) (2020) 11:23. doi: 10.3389/fendo.2020.00023 32117054 PMC7011190

[24] Hu H Nakagawa T Honda T Yamamoto S Okazaki H Yamamoto M. Low Serum Creatinine and Risk of Diabetes: The Japan Epidemiology Collaboration on Occupational Health Study. J Diabetes Investig (2019) 10(5):1209–14. doi: 10.1111/jdi.13024 PMC6717816 30756513

[25] Knott C Bell S Britton A. Alcohol Consumption and the Risk of Type 2 Diabetes: A Systematic Review and Dose-Response Meta-Analysis of More Than 1.9 Million Individuals From 38 Observational Studies. Diabetes Care (2015) 38(9):1804–12. doi: 10.2337/dc15-0710 26294775

[26] Stower H. Smoking Cessation and Type 2 Diabetes. Nat Med (2020) 26(2):163. doi: 10.1038/s41591-020-0779-6 32047321

[27] White IR Royston P Wood AM. Multiple Imputation Using Chained Equations: Issues and Guidance for Practice. Stat Med (2011) 30(4):377–99. doi: 10.1002/sim.4067 21225900

[28] Groenwold RH White IR Donders AR Carpenter JR Altman DG Moons KG. Missing Covariate Data in Clinical Research: When and When Not to Use the Missing-Indicator Method for Analysis. CMAJ (2012) 184(11):1265–9. doi: 10.1503/cmaj.110977 PMC3414599 22371511

[29] Appropriate Body-Mass Index for Asian Populations and its Implications for Policy and Intervention Strategies. Lancet (2004) 363(9403):157–63. doi: 10.1016/S0140-6736(03)15268-3 14726171

[30] Heijmans N van Lieshout J Wensing M. Social Network Composition of Vascular Patients and its Associations With Health Behavior and Clinical Risk Factors. PloS One (2017) 12(9):e0185341. doi: 10.1371/journal.pone.0185341 28957372 PMC5619748

[31] Haneuse S VanderWeele TJ Arterburn D. Using the E-Value to Assess the Potential Effect of Unmeasured Confounding in Observational Studies. JAMA (2019) 321(6):602–3. doi: 10.1001/jama.2018.21554 30676631

[32] von Elm E Altman DG Egger M Pocock SJ Gøtzsche PC Vandenbroucke JP. The Strengthening the Reporting of Observational Studies in Epidemiology (STROBE) Statement: Guidelines for Reporting Observational Studies. Int J Surg (2014) 12(12):1495–9. doi: 10.1016/j.ijsu.2014.07.013 25046131

[33] Heianza Y Hara S Arase Y Saito K Fujiwara K Tsuji H. HbA1c 5·7-6·4% and Impaired Fasting Plasma Glucose for Diagnosis of Prediabetes and Risk of Progression to Diabetes in Japan (TOPICS 3): A Longitudinal Cohort Study. Lancet (2011) 378(9786):147–55. doi: 10.1016/S0140-6736(11)60472-8 21705064

[34] Lipska KJ Inzucchi SE Van Ness PH Gill TM Kanaya A Strotmeyer ES. Elevated HbA1c and Fasting Plasma Glucose in Predicting Diabetes Incidence Among Older Adults: Are Two Better Than One? Diabetes Care (2013) 36(12):3923–9. doi: 10.2337/dc12-2631 PMC3836095 24135387

[35] Behiry EG El NN AbdEl HO Mattar MK Magdy A. Evaluation of TG-HDL Ratio Instead of HOMA Ratio as Insulin Resistance Marker in Overweight and Children With Obesity. Endocr Metab Immune Disord Drug Targets (2019) 19(5):676–82. doi: 10.2174/1871530319666190121123535 30663576

[36] Uruska A Zozulinska-Ziolkiewicz D Niedzwiecki P Pietrzak M Wierusz-Wysocka B. TG/HDL-C Ratio and Visceral Adiposity Index may be Useful in Assessment of Insulin Resistance in Adults With Type 1 Diabetes in Clinical Practice. J Clin Lipidol (2018) 12(3):734–40. doi: 10.1016/j.jacl.2018.01.005 29523408

[37] Sung KC Park HY Kim MJ Reaven G. Metabolic Markers Associated With Insulin Resistance Predict Type 2 Diabetes in Koreans With Normal Blood Pressure or Prehypertension. Cardiovasc Diabetol (2016) 15:47. doi: 10.1186/s12933-016-0368-7 27001495 PMC4802716

[38] Cheng C Liu Y Sun X Yin Z Li H Zhang M. Dose-Response Association Between the Triglycerides: High-Density Lipoprotein Cholesterol Ratio and Type 2 Diabetes Mellitus Risk: The Rural Chinese Cohort Study and Meta-Analysis. J Diabetes (2019) 11(3):183–92. doi: 10.1111/1753-0407.12836 30091266

[39] Zhou M Li Z Min R Dong Y Sun Q Li Y. Log (TG)/HDL-C Ratio as a Predictor of Decreased Islet Beta Cell Function in Patients With Type 2 Diabetes: 6-Year Cohort Study. J Diabetes (2015) 7(5):689–98. doi: 10.1111/1753-0407.12229 25327383

[40] Sorci-Thomas MG Thomas MJ. High Density Lipoprotein Biogenesis, Cholesterol Efflux, and Immune Cell Function. Arterioscler Thromb Vasc Biol (2012) 32(11):2561–5. doi: 10.1161/ATVBAHA.112.300135 PMC3793253 23077142

[41] Koren-Gluzer M Aviram M Meilin E Hayek T. The Antioxidant HDL-Associated Paraoxonase-1 (PON1) Attenuates Diabetes Development and Stimulates β-Cell Insulin Release. Atherosclerosis (2011) 219(2):510–8. doi: 10.1016/j.atherosclerosis.2011.07.119 21862013

[42] Shimodaira M Niwa T Nakajima K Kobayashi M Hanyu N Nakayama T. Serum Triglyceride Levels Correlated With the Rate of Change in Insulin Secretion Over Two Years in Prediabetic Subjects. Ann Nutr Metab (2014) 64(1):38–43. doi: 10.1159/000360012 24732283

[43] Siegel LC Sesso HD Bowman TS Lee IM Manson JE Gaziano JM. Physical Activity, Body Mass Index, and Diabetes Risk in Men: A Prospective Study. Am J Med (2009) 122(12):1115–21. doi: 10.1016/j.amjmed.2009.02.008 PMC2789347 19958889

[44] Climie RE van Sloten TT Bruno RM Taddei S Empana JP Stehouwer C. Macrovasculature and Microvasculature at the Crossroads Between Type 2 Diabetes Mellitus and Hypertension. Hypertension (2019) 73(6):1138–49. doi: 10.1161/HYPERTENSIONAHA.118.11769 31067192

[45] Wakabayashi I. Light-To-Moderate Alcohol Drinking Reduces the Impact of Obesity on the Risk of Diabetes Mellitus. J Stud Alcohol Drugs (2014) 75(6):1032–8. doi: 10.15288/jsad.2014.75.1032 25343662

[46] Jaiswal M Schinske A Pop-Busui R. Lipids and Lipid Management in Diabetes. Best Pract Res Clin Endocrinol Metab (2014) 28(3):325–38. doi: 10.1016/j.beem.2013.12.001 24840262

[47] Weng J Ji L Jia W Lu J Zhou Z Zou D. Standards of Care for Type 2 Diabetes in China. Diabetes Metab Res Rev (2016) 32(5):442–58. doi: 10.1002/dmrr.2827 PMC5108436 27464265

