# Supplementary figures and images for: The Association Between the Triglyceride-to-High-Density Lipoprotein Cholesterol Ratio and the Risk of Progression to Diabetes From Prediabetes: A 5-year Cohort Study in Chinese Adults
Source: Front Endocrinol (Lausanne). 2022 Jul 18;13:947157. doi: 10.3389/fendo.2022.947157 (PMC9340202; doi:10.3389/fendo.2022.947157)

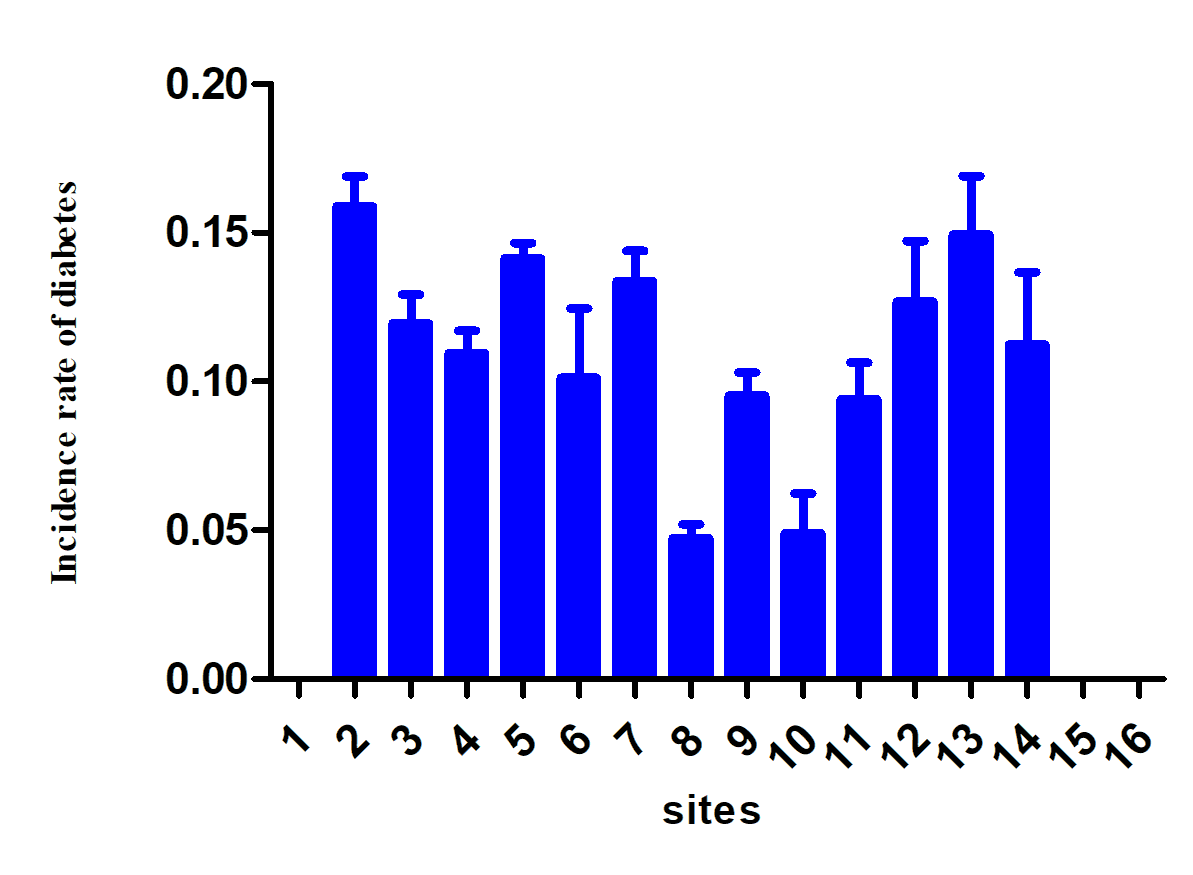

Supplement: Supplementary file 1 [file Image_1.tif]
