# Supplementary material for: The Association Between the Triglyceride-to-High-Density Lipoprotein Cholesterol Ratio and the Risk of Progression to Diabetes From Prediabetes: A 5-year Cohort Study in Chinese Adults
Source: Front Endocrinol (Lausanne). 2022 Jul 18;13:947157. doi: 10.3389/fendo.2022.947157 (PMC9340202; doi:10.3389/fendo.2022.947157)
Supplement: Supplementary file 2 [file Table_1.docx]

**The association between triglyceride-to-high density lipoprotein cholesterol ratio and** **the risk of progression to diabetes from prediabetes: a 5-year cohort study in Chinese adults.**

**Running title:** **TG/HDL-c ratio and diabetes**

Yanfei Sun^1#^, Zhibin Wang^2#^ ,Zhiqiang Huang^2#^, Haofei Hu^3*^, Yong han^2*^

^1^ Department of trauma center/burns, The First Affiliated Hospital of China Medical University, Shenyang, 110001, Liaoning Province, China

^2^ Department of emergency, Shenzhen Second People's Hospital, Shenzhen 518035, Guangdong Province, China

^3^ Department of nephrology, Shenzhen Second People's Hospital, Shenzhen 518035, Guangdong Province, China

Yanfei Sun^1#^, Zhibin Wang^2#^ ,and Zhiqiang Huang^2#^ have contributed equally to this work.

*Corresponding author

Haofei Hu^3*^

Department of nephrology, Shenzhen Second People's Hospital

No.3002 Sungang Road, Futian District,

Shenzhen 518035,

Guangdong Province,

China.

[huhaofei0319@126.com](mailto:huhaofei0319@126.com)

*Corresponding author

Yong Han

Department of emergency, Shenzhen Second People's Hospital

No.3002 Sungang Road, Futian District,

Shenzhen 518035,

Guangdong Province,

China

E-mail: hanyong511023@163.com

**Table S1. Stratified associations between TG/HDL-c ratio and diabetes in participants with prediabetes by age, sex, BMI, SBP,**

| Characteristic | No of participants | HR (95%CI) P value P for interaction |
| --- | --- | --- |
| Age, years  <30  30 to <40  40 to <50  50 to <60  60 to <70 | 589  3047  3387  3818  2916 | 0.083  1.198 (0.640, 2.242) 0.572  1.440 (1.260, 1.647) <0.001  1.247 (1.135, 1.370) <0.001  1.046 (0.964, 1.135) 0.281  1.037 (0.948, 1.135) 0.428 |
| ≥70 | 1350 | 1.030 (0.897, 1.182) 0.680 |
| Sex |  | 0.063 |
| Male | 9745 | 1.094 (1.039, 1.153) <0.001 |
| Female | 5362 | 1.214 (1.103, 1.335) <0.001 |
| BMI (kg/m^2^) |  | 0.519 |
| <18.5 | 257 | 1.276 (0.323, 5.039) 0.728 |
| ≥18.5, < 25 | 7881 | 1.179 (1.092, 1.274) <0.001 |
| ≥25 | 6969 | 1.094 (1.035, 1.157) 0.002 |
| SBP(mmHg) |  | 0.696 |
| <140 | 11735 | 1.108 (1.049, 1.171) <0.001 |
| ≥140 | 3372 | 1.110 (1.026, 1.201) 0.009 |

Note 1: Abolve model adjusted for age, sex, BMI, SBP, DBP, ALT, AST, Scr, family history of diabetes

Note 2: In each case, the model is not adjusted for the stratification variable.

HR, Hazard ratios; CI: confidence, Ref: reference.

**Table S2 The Baseline Characteristics of participants on both sides of the inflection point**

| TG/HD ratio | <1.415 | >=1.415 | Standardize diff. | P-value |
| --- | --- | --- | --- | --- |
| N | 9866 | 5241 |  |  |
| AGE | 50.35 ± 13.87 | 52.07 ± 12.63 | 0.13 (0.10, 0.16) | <0.001 |
| BMI(kg/m^2^) | 24.16 ± 3.25 | 26.00 ± 3.09 | 0.58 (0.55, 0.61) | <0.001 |
| SBP(mmHg) | 126.15 ± 17.80 | 129.86 ± 17.24 | 0.21 (0.18, 0.25) | <0.001 |
| DBP(mmHg) | 77.27 ± 11.17 | 80.63 ± 10.86 | 0.31 (0.27, 0.34) | <0.001 |
| TC(mmol/L) | 4.93 ± 0.92 | 5.21 ± 0.95 | 0.30 (0.26, 0.33) | <0.001 |
| LDL-c(mmol/L) | 2.90 ± 0.69 | 3.02 ± 0.75 | 0.16 (0.12, 0.19) | <0.001 |
| ALT(U/L) | 24.72 ± 21.64 | 33.86 ± 24.26 | 0.40 (0.36, 0.43) | <0.001 |
| AST(U/L) | 24.98 ± 11.27 | 28.45 ± 12.25 | 0.30 (0.26, 0.33) | <0.001 |
| BUN(mmol/L) | 5.01 ± 1.26 | 4.98 ± 1.20 | 0.02 (-0.01, 0.06) | 0.158 |
| sCr(μmol/L) | 71.64 ± 16.03 | 75.44 ± 16.08 | 0.24 (0.20, 0.27) | <0.001 |
| Sex |  |  | 0.38 (0.35, 0.42) | <0.001 |
| Male | 5758 (58.36%) | 3987 (76.07%) |  |  |
| Fmeale | 4108 (41.64%) | 1254 (23.93%) |  |  |
| Smoking status |  |  | 0.26 (0.23, 0.29) | <0.001 |
| Current smoker | 1766 (17.90%) | 1500 (28.62%) |  |  |
| Ever smoker | 379 (3.84%) | 224 (4.27%) |  |  |
| Never smoker | 7721 (78.26%) | 3517 (67.11%) |  |  |
| Drinking status |  |  | 0.16 (0.13, 0.20) | <0.001 |
| Current drinker | 291 (2.95%) | 284 (5.42%) |  |  |
| Ever drinker | 1536 (15.57%) | 998 (19.04%) |  |  |
| Never drinker | 8039 (81.48%) | 3959 (75.54%) |  |  |
| Family history of diabetes |  |  | 0.01 (-0.03, 0.04) | 0.751 |
| No | 9611 (97.42%) | 5101 (97.33%) |  |  |
| Yes | 255 (2.58%) | 140 (2.67%) |  |  |

Values are n (%) or mean ± SD or median (quartile)

TC, total cholesterol; LDL-c, low-density lipoproteins cholesterol; BUN, blood urea nitrogen; **sCr** serum creatinine; ALT, alanine aminotransferase; AST, aspartate aminotransferase; DBP, diastolic blood pressure; SBP, systolic blood pressure; TG/HDL-C ratio, triglyceride-to-high density lipoprotein cholesterol ratio.

**Table S3 The Baseline Characteristics of** **the three populations with normoglycaemia, still in the prediabetic stage and diagnosed diabetes**

|  | normoglycaemia | prediabetes | diabetes | P-value |
| --- | --- | --- | --- | --- |
| N | 6863 | 6513 | 1731 |  |
| Age(years) | 52.71 ± 12.99 | 47.48 ± 13.37 | 56.97 ± 12.30 | <0.001 |
| BMI(kg/m^2^) | 25.07 ± 3.22 | 24.19 ± 3.28 | 26.03 ± 3.34 | <0.001 |
| SBP(mmHg) | 129.31 ± 17.69 | 124.12 ± 16.94 | 132.49 ± 18.22 | <0.001 |
| DBP(mmHg) | 79.54 ± 11.20 | 76.72 ± 10.86 | 80.51 ± 11.40 | <0.001 |
| TC(mmol/L) | 5.06 ± 0.93 | 4.97 ± 0.93 | 5.10 ± 0.96 | <0.001 |
| TG(mmol/L) | 1.72 ± 1.01 | 1.55 ± 0.95 | 1.99 ± 1.15 | <0.001 |
| HDL-c(mmol/L) | 1.33 ± 0.29 | 1.36 ± 0.29 | 1.31 ± 0.36 | <0.001 |
| TG/HDL-c ratio | 1.39 ± 0.93 | 1.24 ± 0.89 | 1.64 ± 1.04 | <0.001 |
| LDL-c(mmol/L) | 2.97 ± 0.71 | 2.92 ± 0.72 | 2.95 ± 0.71 | <0.001 |
| ALT(U/L) | 28.28 ± 24.44 | 25.84 ± 19.59 | 34.04 ± 27.41 | <0.001 |
| AST(U/L) | 26.43 ± 12.47 | 25.13 ± 10.19 | 29.15 ± 13.49 | <0.001 |
| BUN(mmol/L) | 5.05 ± 1.23 | 4.94 ± 1.24 | 5.05 ± 1.27 | <0.001 |
| sCr (μmol/L) | 73.79 ± 15.88 | 72.02 ± 16.29 | 73.21 ± 16.52 | <0.001 |
| Sex |  |  |  | <0.001 |
| Male | 4571 (66.60%) | 3934 (60.40%) | 1240 (71.63%) |  |
| Fmeale | 2292 (33.40%) | 2579 (39.60%) | 491 (28.37%) |  |
| Smoking status |  |  |  | <0.001 |
| Never smoker | 5023 (73.19%) | 5060 (77.69%) | 1155 (66.72%) |  |
| Ever smoker | 260 (3.79%) | 245 (3.76%) | 98 (5.66%) |  |
| Current smoker | 1580 (23.02%) | 1208 (18.55%) | 478 (27.61%) |  |
| Drinking status |  |  |  | <0.001 |
| Never drinker | 5407 (78.78%) | 5212 (80.02%) | 1379 (79.66%) |  |
| Ever drinker | 1155 (16.83%) | 1109 (17.03%) | 270 (15.60%) |  |
| Current drinker | 301 (4.39%) | 192 (2.95%) | 82 (4.74%) |  |
| Family history of diabetes |  |  |  | <0.001 |
| No | 6707 (97.73%) | 6347 (97.45%) | 1658 (95.78%) |  |
| Yes | 156 (2.27%) | 166 (2.55%) | 73 (4.22%) |  |

**Figure S1 Epidemiological distribution of diabetes prevalence in participants with prediabetes at 16 sites**
